# Targeting glycolysis in esophageal squamous cell carcinoma: single-cell and multi-omics insights for risk stratification and personalized therapy

**DOI:** 10.3389/fphar.2025.1559546

**Published:** 2025-03-06

**Authors:** Yan Wang, Yunjie Shi, Xiao Hu, Chenfang Wang

**Affiliations:** ^1^ Department of Anesthesia, First Affiliated Hospital of Chengdu Medical College, Chengdu, Sichuan, China; ^2^ School of Clinical Medicine, Chengdu Medical College, Chengdu, Sichuan, China

**Keywords:** esophageal squamous cell carcinoma, glycolysis, tumor microenvironment, risk score model, immune microenvironment

## Abstract

**Background:**

Esophageal squamous cell carcinoma (ESCC) is closely linked to aberrant glycolytic metabolism, a hallmark of cancer progression, immune evasion, and therapy resistance. This study employs single-cell transcriptomics and multi-omics approaches to unravel glycolysis-mediated mechanisms in ESCC, with a focus on risk stratification and therapeutic opportunities.

**Methods:**

Data from TCGA and GEO databases were integrated with single-cell RNA sequencing, bulk RNA sequencing, as well as clinical datasets to investigate glycolysis-associated cell subtypes and their clinical implications in ESCC. Analytical approaches encompassed cell subtype annotation, cell-cell communication network analysis, and gene regulatory network modeling. A glycolysis-related risk score model was built via non-negative matrix factorization (NMF) and Cox regression, and then experimentally verified through Western blotting. Drug sensitivity analyses were carried out to explore potential therapeutic strategies.

**Results:**

Single-cell analysis identified epithelial cells as the dominant glycolysis-active subtype, and tumor tissues showed significantly higher glycolytic activity than adjacent normal tissues. Among malignant epithelial subpopulations, IGFBP3+Epi (IGFBP3-expressing epithelial cells) and LHX9+Epi (LHX9-expressing epithelial cells) had elevated glycolysis levels, which correlated with poor prognosis, immune suppression, and changes in the tumor microenvironment. The seven-gene glycolysis-based risk score model divided patients into high- and low-risk groups, demonstrating strong prognostic performance. Drug sensitivity analysis showed high-risk patients were more responsive to Navitoclax as well as Rapamycin, but low-risk ones were more sensitive to Afatinib and Erlotinib, highlighting the model’s usefulness in guiding personalized treatment.

**Conclusion:**

This research emphasizes the crucial role of glycolysis in ESCC progression a well as immune modulation, offering a novel glycolysis-related risk score model with significant prognostic and therapeutic implications. These findings provide a basis for risk-based stratification and tailored therapeutic strategies, advancing precision medicine in ESCC.

## 1 Introduction

Esophageal cancer (EC) is the sixth most prevalent cause of cancer-related deaths worldwide and the eighth most common type of cancer overall (Uhlenhopp et al., 2020). Projections show by 2040, the number of global EC cases will increase to 913,300, with 867,400 deaths. The incidence and histological characteristics of EC vary significantly across different regions. In the US, the diagnosis rate of esophageal cancer is relatively low, with an estimated 21,560 new cases as well as 16,120 deaths expected in 2023 (Siegel et al., 2023). In contrast, China reported 224,000 new cases of esophageal cancer and 187,500 deaths in 2022, accounting for 4.64% and 7.28% of all malignant tumors, respectively (The Medical Emergency Division of the National Health Commission, 2024). Esophageal cancer mainly consists of two histological categories, namely squamous cell carcinoma (SCC) as well as adenocarcinoma (AC). Among them, esophageal squamous cell carcinoma (ESCC) is the most prevalent subtype, making up around 90% of all cases (Song et al., 2014). Approximately 500,000 new cases of ESCC are diagnosed annually, with a high mortality rate of 90% (Sung et al., 2021). More than half of ESCC cases occur in China, where it is the sixth most common cause of death due to cancer worldwide (Chen et al., 2017). Despite advancements in diagnostic as well as treatment technologies in th past years s, the 5-year survival rate for ESCC patients remains only 12%–20% (Napier et al., 2014). The high incidence and poor prognosis of ESCC highlight the urgent need to improve diagnostic and therapeutic strategies. Currently, the main treatments for ESCC include surgery, chemotherapy, as well as radiation therapy. However, due to the highly invasive nature of ESCC, many individuals are diagnosedwhen they are at advanced stages, limiting the effectiveness of these treatments (Shimizu et al., 2023). Therefore, in-depth research into the biological mechanisms of ESCC, particularly the molecular mechanisms related to metabolic pathways, is important for comprehending disease progression as well as improving patient prognosis.

The occurrence, development, and metastasis of tumors have a close bearing on metabolic pathways, with metabolic reprogramming playing a key role in these processes (Martínez-Reyes and Chandel, 2021; Pavlova and Thompson, 2016). Recent studies have shown that ESCC patients often exhibit significant disruptions in circulating metabolites (Tao et al., 2021; Chen et al., 2020). In the metabolic processes of ESCC, the distribution of certain key metabolites undergoes significant changes, and these metabolites are widely involved in important metabolic pathways such as glycolysis, anaerobic respiration, the tricarboxylic acid (TCA) cycle, as well as protein and lipid metabolism (Huang et al., 2020). Research by Yang et al. has shown that knocking out the Sirt1 gene can regulate the glycolytic pathway, thereby enhancing the sensitivity of ESCC to chemotherapy (Yang X. et al., 2024). Han et al. further discovered that peripheral coumarin (PPM) can simultaneously regulate glycolysis and mitochondrial oxidative phosphorylation (OXPHOS), inhibiting the proliferation of ESCC cells *in vitro*, inducing apoptosis, and causing cell cycle arrest at the G2/M phase (Han et al., 2024). Additionally, research shows inhibiting glycolysis and mitochondrial oxidative phosphorylation at the same time results in fatal energy depletion, which effectively stops tumor growth. (Dong et al., 2022). Moreover, key glycolytic enzymes are commonly overexpressed in ESCC. For example, hexokinase 2 (HK2), pyruvate kinase M2 (PKM2), and lactate dehydrogenase A (LDHA) are remarkably improved in ESCC patients. The abnormal expression of these enzymes not only promotes glycolytic activity but is also closely associated with poor clinical prognosis (Yang et al., 2021). Thus, glycolysis has a profound impact on the tumor biology of ESCC, making it a current hot research topic. Research into the molecular mechanisms of the glycolytic pathway provides fresh insights into the pathogenesis of ESCC and lays an important foundation for developing precise and effective therapeutic strategies.

Glycolytic reprogramming is one of the most important metabolic reprogramming processes in tumor cells and is irreplaceable in tumor initiation as well as progression. Glycolytic reprogramming gives tumor cells the energy they need to proliferate quickly, but it also aids in the tumor cells’ adaptation to hypoxic conditions, which increases their chances of surviving and metastasis. As early as the 1920s, Otto Warburg discovered that tumor cells preferentially use glycolysis as their primary energy metabolism pathway, even in the presence of sufficient oxygen, rather than the more efficient oxidative phosphorylation (Thompson et al., 2023; Warburg, 1956). This abnormal glycolytic pathway is known as the “Warburg effect,” which provides tumor cells with macromolecular precursors and an optimal redox environment, meeting the energy demands for growth and division. This process is regarded an adaptive mechanism of tumor cells (Fukushi et al., 2022; Madhukar et al., 2015; Vander Heiden et al., 2009). To enhance antioxidant capacity, various signaling pathways are involved in the glycolysis regulation in tumor cells. For instance, during glycolysis, the tumor suppressor gene p53 can reverse the Warburg effect by inhibiting pro-apoptotic factors, thereby reducing the accumulation of fructose-1,6-bisphosphate (F16BP). Additionally, p53 can upregulate the activity of hexokinase (HK) and phosphoglycerate mutase (PGAM), further enhancing glycolysis under certain conditions (Li et al., 2021). Another example is the IL-17A-HIF1α signaling axis, which can guide the metabolic reprogramming of damaged epithelial cells towards glycolysis, promoting cell migration and tumor metastasis (Konieczny et al., 2022). Moreover, glycolytic reprogramming has a close bearng on the tumor microenvironment (TME). In the low-glucose environment created by tumor cells consuming glucose, regulatory T cells (Tregs) actively absorb lactate (LA) through monocarboxylate transporter 1 (MCT1), promoting the translocation of nuclear factor of activated T-cells 1 into the nucleus and enhancing the expression of programmed cell death protein 1 (PD-1). Conversely, PD-1 expression in effector T cells is suppressed. Blockade of PD-1 may activate Tregs expressing PD-1, thus reducing therapeutic efficacy. It is hypothesized that in a highly glycolytic tumor microenvironment, lactate may be a key regulator of Treg function, upregulating PD-1 expression and forming an active immune checkpoint that suppresses the immune response (Kumagai et al., 2022). Furthermore, glycolytic reprogramming not only affects the metabolism of tumor cells but also weakens the function of immune cells through metabolic competition. For instance, tumor cells can competitively uptake glucose, inhibiting the glycolytic activity of effector T cells as well as NK cells, thereby significantly impairing their anti-tumor capabilities (Chang et al., 2015). In summary, glycolytic reprogramming plays a central part in tumor cell energy supply, biosynthesis, and regulation of the tumor microenvironment. In-depth investigation of its molecular mechanisms and signaling networks will not only reveal the intrinsic nature of tumor metabolic adaptation but also provide new insights and potential targets for cancer therapy.

Single-cell sequencing is a high-resolution analytical technique allowing for the precise analysis of the genome, transcriptome, or epigenome of individual cells, offering significant advantages over traditional bulk sequencing. First, it overcomes the issue of masking individual differences caused by cellular heterogeneity in bulk sequencing, enabling a precise understanding of intercellular heterogeneity and the characteristics of rare cell populations. Second, single-cell sequencing captures the dynamic changes of cells, providing insights into cellular state transitions during development, differentiation, and disease progression, particularly in areas such as tumor evolution, immune responses, and tissue regeneration. Furthermore, this technology enables cell lineage tracing and the construction of cellular maps of complex tissues or organs, allowing for an in-depth analysis of their spatial structure and function. By integrating multi-omics technologies, such as combining single-cell transcriptomics with epigenomics, single-cell sequencing can explore the biological properties of cells from multiple dimensions, providing powerful tools for both basic research and precision medicine.

In this study, single-cell sequencing technology precisely captured the glycolytic characteristics of different cell subtypes within the tumor microenvironment, revealing the metabolic differences between ESCC cell subpopulations and their role in tumor progression. Additionally, a glycolysis-related risk scoring model was constructed through multi-omics analysis, systematically evaluating the association between glycolytic metabolism, patient prognosis, as well as the immune microenvironment. The mode effectively predicts patient survival rates and provides potential strategies for targeting glycolytic metabolism and improving the tumor immune microenvironment. Finally, drug sensitivity analysis was performed, enabling the risk scoring model to guide the selection of chemotherapy drugs, thereby playing a crucial role in precision diagnosis and treatment. Through the integration of single-cell sequencing and multi-omics analysis, this research lays a solid foundation for an in-depth understanding of the metabolic mechanisms of ESCC as well as for personalized treatment approaches.

## 2 Method

### 2.1 Data collection and organization

The single-cell RNA sequencing (scRNA-seq) dataset GSE188900 was downloaded from the GEO database (http://www.ncbi.nlm.nih.gov/geo), including 8 ESCC tissue samples as well as 1 adjacent non-cancerous tissue sample. Additionally, bulk RNA-seq data as well as clinical information from ESCC patients were downloaded from the GEO database (GSE23400, GSE53624, GSE53625). A set of 50 signature gene sets, including the glycolysis-related dataset (HALLMARK_GLYCOLYSIS), was downloaded from the Molecular Signatures Database (MSigDB, http://software.broadinstitute.org/gsea/msigdb/).

### 2.2 Visualization of cell types and subtypes in ESCC

We used the Seurat package (v5.0.1) in R to create a Seurat object. Next, quality control (QC) was performed on the Seurat object with the following filtering criteria: mitochondrial gene percentage below 25%, ribosomal gene percentage above 3%, and red blood cell gene percentage below 5%. After quality control, the functions NormalizeData, FindVariableFeatures, ScaleData, and RunPCA were sequentially applied to compute and obtain the principal components based on the Seurat object. Subsequently, the t-SNE (t-distributed stochastic neighbor embedding) approach was leveraged for dimensionality reduction to better visualize the main principal components. Finally, cell types and their subtypes were annotated and visualized based on the SingleR algorithm and the classic marker genes expressed by each subset.

### 2.3 Cell-cell communication analysis between cell subtypes/clusters

CellChat (PMID: 33597522) is an R package designed to analyse cell-cell communication networks in scRNA-seq data, where different cell populations are labeled. It includes ligand-receptor interaction databases for both humans and mice. First, we used CellChatDB.human to evaluate the major incoming and outgoing signals for each cell subtype/cluster. Then, using the netVisual_circle function, we visualized the cell-cell communication network between cell subtypes/clusters, reflecting the communication intensity from target cell subtypes/clusters to different cell subtypes/clusters. Lastly, using the netVisual_bubble function, we generated a bubble plot to highlight key ligand-receptor interactions between the target cell subtype/cluster and other cell subtypes/clusters.

### 2.4 Single-sample gene set enrichment analysis (ssGSEA) and gene set enrichment analysis (GSEA)

The ssGSEA score for each sample mirrors the extent to which specific gene sets are systematically upregulated or downregulated in that sample. This research used the ssGSEA method from the R package “GSVA” (PMID: 23323831) to obtain glycolysis scores for each sample. To identify pathways related with the characteristics, this research computed the GSVA scores for 50 signature pathways and used the “limma” package to analyze pathways that displayed remarkable variations between the high-risk and low-risk groups. And to uncover biological processes, cellular components, as well as molecular functions involved in various risk subgroups, GSEA analysis was carried out on the GO gene sets (c5. go.v7.5.1. symbols.gmt) between the two risk groups via the “clusterProfiler” R package, applying the recommended thresholds of FDR < 0.25 and |NES| > 1.

### 2.5 Non-negative matrix factorization (NMF) of cell subpopulations with high glycolysis scores in ESCC

To explore the effect of glycolysis on cells within the ESCC tumor microenvironment, we applied the Non-negative Matrix Factorization (NMF) algorithm to perform dimensionality reduction on the expression data of key regulatory factors in immune cells. Next, based on the scRNA-seq expression matrix, this research identified different cell subtypes within these cell populations. All of these analytical steps followed the methods outlined in previous research (PMID: 32686767).

### 2.6 KEGG enrichment and metabolic activity analysis of glycolysis-related cell subtypes in ESCC

To identify biological pathways specific to glycolysis-related cell subtypes in ESCC, this research first employed the FindAllMarkers function to identify differentially expressed genes from the single-cell data, setting the log fold change threshold (logfc.threshold) to 0.25 and the minimum percentage (min.pct) to 0.25. Based on the differentially expressed genes for each cell subtype, we performed KEGG enrichment analysis for each subtype independently and visualized the top three significantly enriched KEGG pathways using heatmaps created with the ggplot2 package. The assessment of metabolic activity was performed using the scMetabolism package (PMID: 34417225). During the analysis, we specifically focused on KEGG metabolic pathways, obtaining activity scores for each metabolic pathway in the cells.

### 2.7 SCENIC analysis

In this study, we used the SCENIC method (PMID: 28991892) to explore single-cell transcriptional regulatory networks. We identified transcription start sites and constructed gene regulatory networks in the ESCC single-cell RNA-seq data based on two gene loci rankings from the RcisTarget database (hg19-tss-centered-10 kb as well a hg19-500 bp-upstream). This approach was used to reveal changes in cell states and transcriptional regulatory mechanisms.

### 2.8 Cox regression analysis of glycolysis-related cell subtype features in ESCC

This research employed the FindAllMarkers function from the Seurat R package to generate gene features for different glycolysis-related cell subtypes in ESCC. These gene features were then used to calculate enrichment scores across all publicly available ESCC datasets using the GSVA function. A Cox regression analysis was carried out to investigate the connection between glycolysis-related cell subtypes in ESCC and patient overall survival (OS).

### 2.9 Identification of glycolysis-related prognostic feature genes in ESCC epithelial cells

The TCGA-ESCC cohort was split into a training cohort as well as a validation cohort at a 7:3 ratio, with the GSE53624 cohort used as an external validation dataset. LASSO Cox analysis was performed via the R packages “glmnet” as well as “survival” to identify prognostic-related glycolysis genes in epithelial cells and to construct a risk score model: Risk score = Σβi αi, where αi and βi denote the expression levels as well as coefficients of the glycolysis genes in the prognostic model. Based on the optimal cutoff from the survival curves, the training, validation cohorts, and GSE53624 cohort were split into high-risk as well as low-risk groups. GSE53624 acted as the external validation dataset. Then, Kaplan-Meier survival curve analysis as well as ROC curve analysis were performed on the training, validation cohorts, and GSE53624 cohort to evaluate the predictive performance of the prognostic glycolysis feature genes in epithelial cells. The R package “timeROC” was employed to plot ROC curves for 1-year, 3-year, as well as 5-year survival. K-M survival curves were plotted via the “survival” as well as “survminer” R packages.

### 2.10 Construction of the nomogram

Combining clinical characteristic data and RiskScore, univariate and multivariate Cox regression analyses were performed to identify prognostic factors, which were subsequently used to construct a Nomogram. The analysis and plotting were conducted using the “rms” package, resulting in a Nomogram to predict the 1-year, 3-year, and 5-year overall survival (OS) rates of patients with early-stage lung adenocarcinoma. The predictive capability of the model was validated using calibration curves and time-dependent ROC curves.

### 2.11 Immune microenvironment analysis

This research employed the R package “IOBR” (PMID: 34276676) to calculate immune cell infiltration scores for 8 immune cell types and analyse the variations in immune-related feature sets between high and low expression groups. Additionally, based on the cell infiltration scores obtained from the CIBERSORT algorithm, we evaluated the impact of various cell gene states on the overall survival (OS) of ESCC patients.

### 2.12 Western blot analysis

HET-1A (ATCC^®^ CRL-2692™, an SV40 T-antigen-immortalized human epithelial cell line originally reported to be derived from normal human esophageal squamous epithelium), KYSE30, and KYSE450 (esophageal cancer cell lines) were purchased from Fuheng Biotechnology (Shanghai, China). HET-1A, KYSE30 and KYSE450 cells were cultured in RPMI-1640 with 10% fetal bovine serum as well as 1% penicillin/streptomycin. All cells were maintained at 37°C with 5% CO_2_. Total protein was extracted using RIPA buffer (50 mM Tris-HCl, pH 7.4, 150 mM NaCl, 1% Triton X-100, 0.5% sodium deoxycholate, 0.1% SDS, and 1 mM EDTA) supplemented with protease and phosphatase inhibitors. Protein concentration was determined using the BCA assay (Thermo Fisher, United States). Equal amounts of protein (30 µg) were separated by 12% SDS-PAGE and transferred to PVDF membranes. Membranes were blocked with 5% non-fat milk and incubated overnight at 4°C with primary antibodies (NFKBIZ, SAB4301779, SAB Biotech, United States; ATF3, ab207434, Abcam, UK; BTG2, ab85051, Abcam; BIK, ab52182, Abcam; IGFBP2, ab109284, Abcam; LY6K, ab246486, Abcam; GAPDH, ab8245, Abcam) diluted 1:1,000. After washing, HRP-conjugated secondary antibody was applied, and protein bands were visualized using ECL substrate (Thermo Fisher). Image analysis was performed using the Chemidoc XRS+ system (Bio-Rad, United States). GAPDH was employed as a loading control.

### 2.13 Statistical analysis

All statistical analyses in this study were carrried out using R 4.3.2. The t-test, Wilcoxon rank-sum test, chi-square test, and Kruskal-Wallis test were leveraged to assess differences in continuous or categorical variables between different cell groups. A p-value < 0.05 was regarded statistically significant.

## 3 Result

### 3.1 Overview of Glycolysis in ESCC single-cell data

**FIGURE 1:**
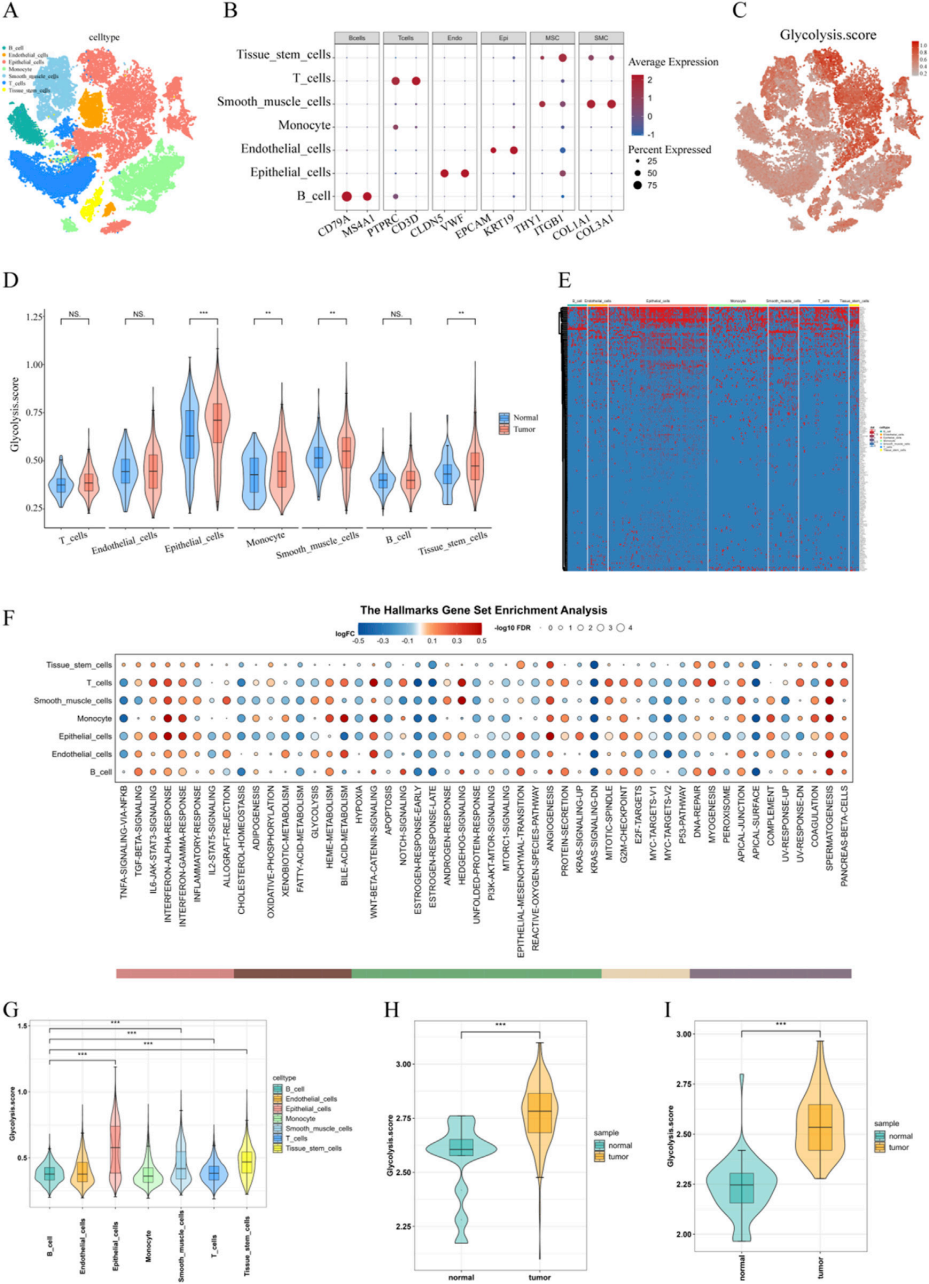
Overview of Glycolysis in ESCC Single-Cell Data. **(A)** t-SNE plot of cell subpopulation classification; **(B)** Bubble plot of classical marker gene expression for each cell subpopulation; **(C)** t-SNE plot of glycolysis scores for each cell subtype; **(D)** Glycolysis scores for each cell subtype between cancer and adjacent non-cancerous tissues; **(E)** Heatmap of glycolysis gene expression across cell subtypes; **(F)** GSVA scores for 50 hallmark gene sets across each cell subtype; **(G)** Quantitative violin plot of glycolysis scores for each cell subtype; **(H)** Glycolysis scores for cancer and adjacent non-cancerous tissues in the TCGA-ESCA cohort; **(I)** Glycolysis scores for cancer and adjacent non-cancerous tissues in the GSE23400 cohort.

We used the GSE188900 scRNA-seq dataset to study the glycolytic state of major cell subtypes in ESCC. This dataset includes 8 ESCC tissue samples and 1 adjacent non-cancerous tissue sample. The major cell subpopulations, like T cells, B cells, epithelial cells, endothelial cells, monocytes, smooth muscle cells, as well as tissue stem cells, were annotated using SingleR (Figure 1A). Additionally, the accuracy of the annotations was confirmed based on the expression of typical marker genes, and a bubble plot was used to display the expression levels and proportions of specific markers for each cell type (Figure 1B). Furthermore, the GSVA scores for 50 hallmark gene sets (HALLMARK) for each cell subtype were also presented in a bubble plot (Figure 1F). As shown in Figure 1E, glycolysis gene expression varies across different cell subtypes, with epithelial cells showing elevated glycolytic activity compared to other cell types. To explore the glycolytic state of different cell types, this research employed the ssGSEA algorithm to assess the glycolysis score for each cell in the samples, and the results were mapped onto a t-SNE plot. The plot shows that epithelial cells have significantly higher glycolysis scores compared to other cell types (Figures 1C, G). Notably, glycolysis scores were higher in cancer tissues compared to adjacent normal tissues (Figures 1H, I). Moreover, epithelial cells, monocytes, smooth muscle cells, and tissue stem cells in cancer tissues exhibited higher glycolysis scores (Figure 1D).

### 3.2 Features of ESCC epithelial cells and glycolysis-mediated characteristics of related epithelial cell subtypes

**FIGURE 2:**
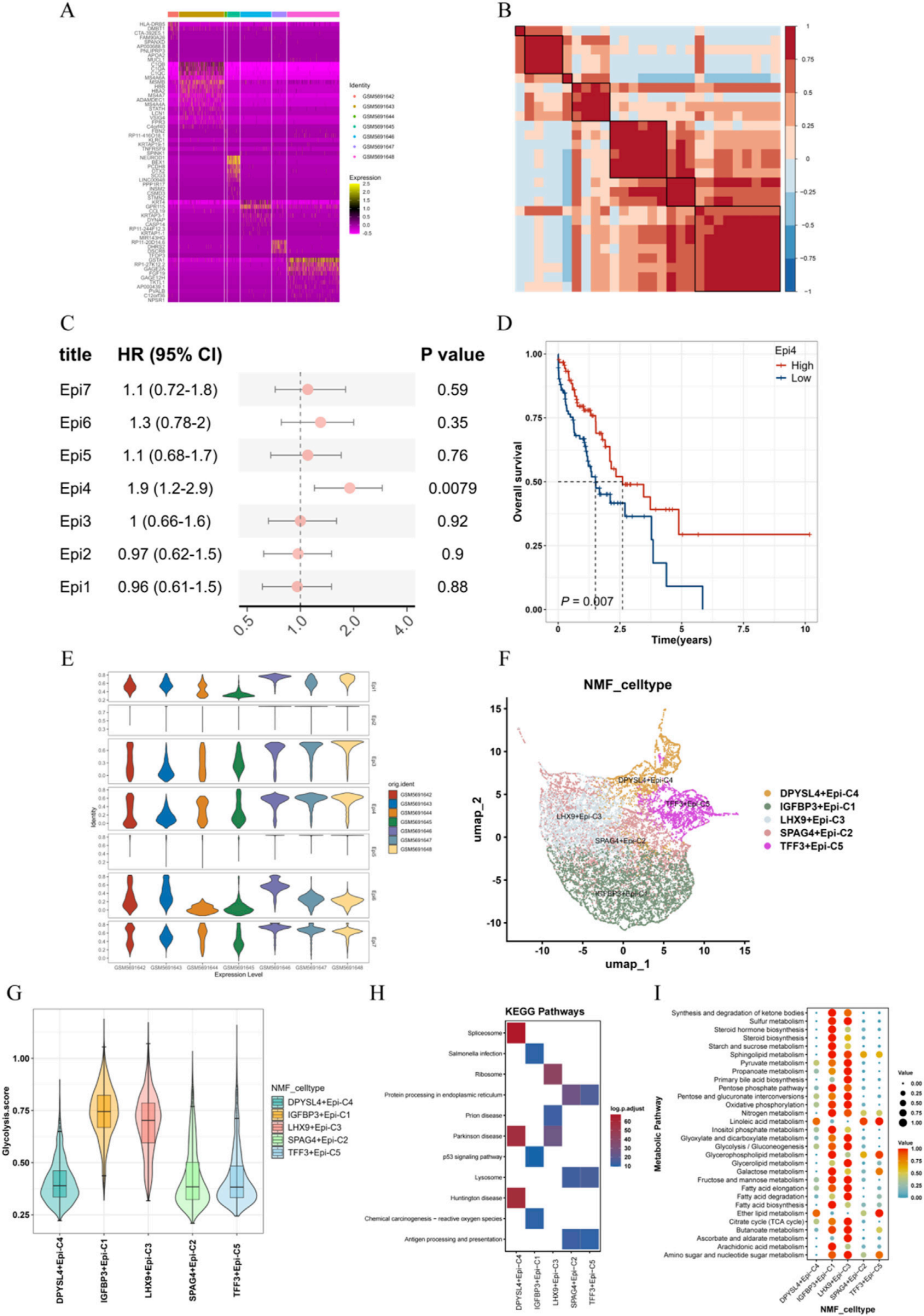
Features of ESCC Epithelial Cells and Glycolysis-Mediated Characteristics of Related Epithelial Cell Subpopulations. **(A)** Heatmap of epithelial cell marker genes across different samples; **(B)** NMF clustering correlation heatmap; **(C)** Univariate Cox regression forest plot for epithelial cell features in the TCGA-ESCA cohort; **(D)** Survival curve for high and low Epi4 feature scores in the TCGA-ESCA cohort; **(E)** GSVA scores for epithelial cell features across different samples; **(F)** NMF cell clustering plot; **(G)** Glycolysis scores of newly defined glycolysis-related cell subpopulations; **(H)** KEGG enrichment result plot; **(I)** Metabolic activity analysis result plot.

There may be differences between malignant epithelial cells from different sample sources. To assess these differences, we calculated the differential genes in epithelial cells from various samples and plotted a heatmap. As shown in Figure 2A, malignant epithelial cells from different sample sources indeed exhibit distinct features. To identify consistent genes between malignant cells across different samples, we carried out NMF dimensionality reduction analysis on glycolysis genes to identify core genes that are preferentially co-expressed in malignant cell subpopulations across different tumors. These core genes were then characterized as gene expression features through hierarchical clustering, with 7 features showing high consistency (Figure 2B). Malignant feature scores for malignant cells from different tumors varied (Figure 2E), but univariate Cox analysis based on the TCGA-ESCA cohort indicated that among these 7 malignant features, Epi4 may serve as a risk factor influencing patient prognosis in ESCC (Figure 2C). The Kaplan-Meier (KM) curve results were consistent with the Cox regression results (Figure 2D). To further investigate the impact of glycolysis on malignant epithelial cells, we mapped the NMF clustering results onto a UMAP plot, identifying 5 glycolysis-related malignant cell subpopulations (Figure 2F). Among these newly defined subpopulations, the IGFBP3+Epi and LHX9+Epi subpopulations showed significantly higher glycolysis scores than the other subpopulations (Figure 2G). KEGG enrichment results revealed that these subpopulations were mainly associated with ribosome, lysosome, and p53 pathways (Figure 2H). The metabolic activity of different subpopulations was assessed, as shown in Figure 2I, where the metabolic characteristics of glycolysis-related epithelial cell subpopulations were analyzed.

### 3.3 Analysis of ESCC T cell features based on glycolysis-related regulatory factors

**FIGURE 3:**
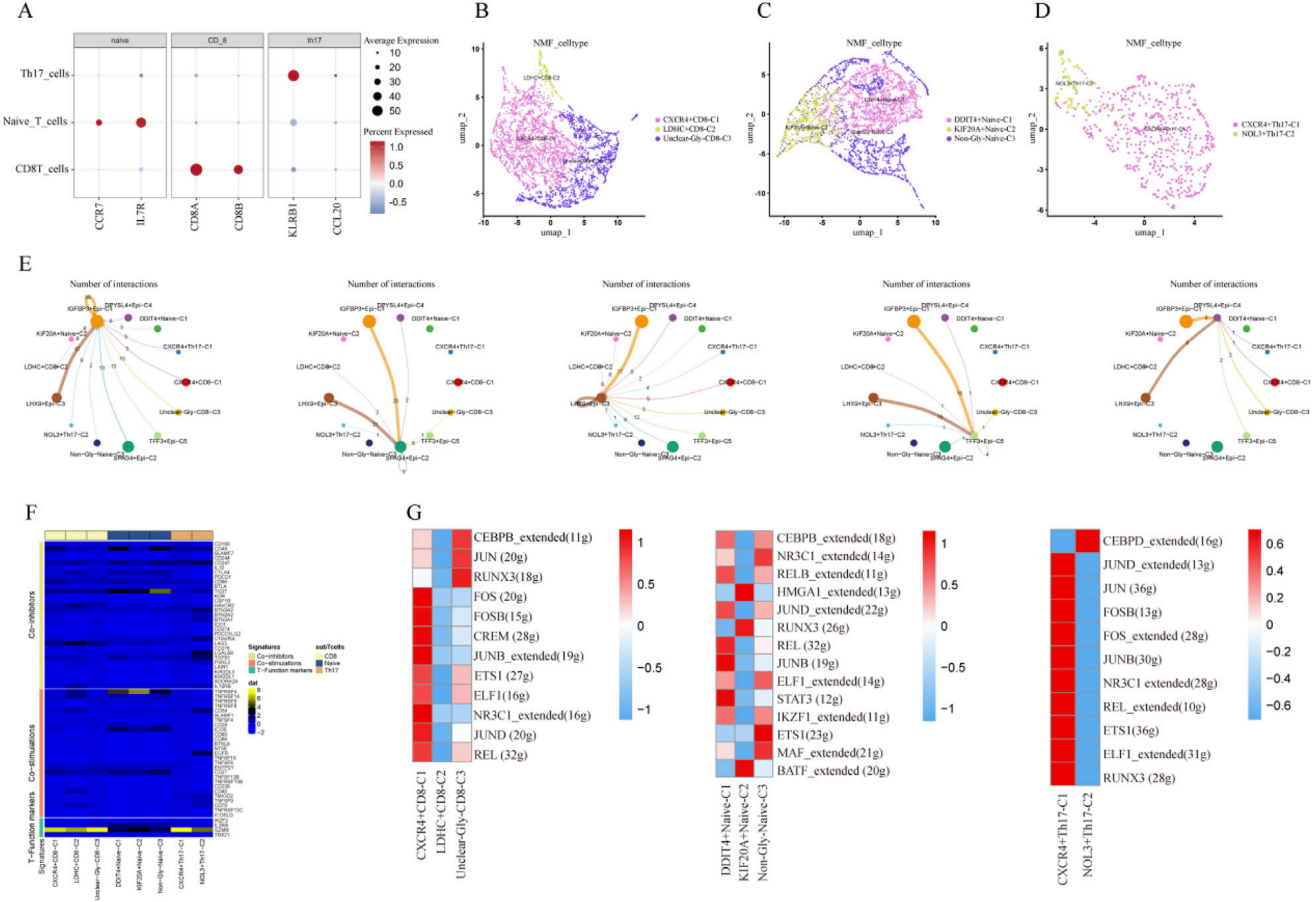
Analysis of ESCC T Cell Features Based on Glycolysis-Related Regulatory Factors. **(A)** Bubble plot of classical marker gene expression for T cell subpopulations; **(B)** NMF clustering plot of CD8^+^ T cells; **(C)** NMF clustering plot of Th17 cells; **(D)** NMF clustering plot of Naïve T cells; **(E)** Cell communication between various T cell subpopulations and newly defined glycolysis-mediated epithelial cells; **(F)** Heatmap showing the expression of immune stimulatory factors, inhibitory factors, as well as T cell functional marker genes in CD8^+^, Th17, and Naïve cells; **(G)** Heatmap of transcription factor activity in CD8^+^, Th17, and Naïve T cells.

Based on the classical markers of T cells, we identified three T cell subtypes in this dataset: CD8+ T cells, Th17 cells, as well as Naïve T cells (Figure 3A). Using NMF clustering, we defined new T cell subtypes, including CXCR4+CD8, LDHC+CD8, Unclear-Gly-CD8, NOL3+Th17, CXCR4+Th17, DDIT4+Naïve, KIF20A+Naïve, and Unclear-Gly-Naïve (Figures 3B–D). To explore the impact of these subpopulations on malignant epithelial cells, we carried out cell-cell communication analysis. The findings showed the IGFBP3+Epi (IGFBP3-expressing epithelial cells) and LHX9+Epi (LHX9-expressing epithelial cells) subpopulations, with the highest glycolysis scores, showed communication with all newly defined T cell subpopulations compared to other malignant subpopulations (Figure 3E). And to assess the overall effect of glycolysis-related T cell subpopulations, this research found significant variations in the average expression of immune genes associated with co-stimulation, co-inhibition, as well as functional markers (Figure 3F). To further understand the intercellular regulatory network, this research carried out SCENIC analysis to examine the activity of transcription factors. The results suggested the transcription factor activity in CXCR4+CD8, DDIT4+Naïve, and CXCR4+Th17 T cell subtypes was significantly higher than in other cell types (Figure 3G).

### 3.4 Analysis of ESCC smooth muscle cells and tissue stem cell features based on glycolysis-related regulatory Factors

**FIGURE 4:**
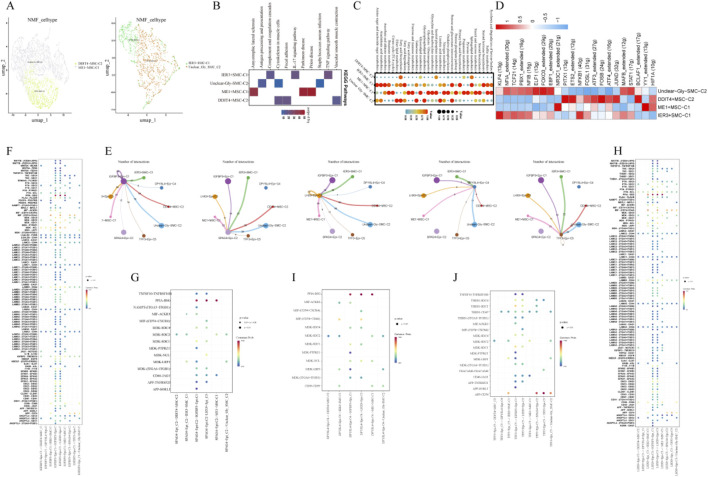
Analysis of ESCC Smooth Muscle Cells and Tissue Stem Cell Features Based on Glycolysis-Related Regulatory Factors. **(A)** NMF clustering results of SMC and MSC cells; **(B)** KEGG enrichment result plot; **(C)** Metabolic activity analysis result plot; **(D)** Transcription factor activity heatmap; **(E)** Cell communication between various SMC, MSC cells and newly defined glycolysis-mediated epithelial cells; **(F)** Ligand-receptor interactions between SMC, MSC cells and IGFBP3+Epi cells; **(G)** Ligand-receptor interactions between SMC, MSC cells and SPAG4+Epi cells; **(H)** Ligand-receptor interactions between SMC, MSC cells and LHX9+Epi cells; **(I)** Ligand-receptor interactions between SMC, MSC cells and DPYSL4+Epi cells; **(J)** Ligand-receptor interactions between SMC, MSC cells and TFF3+Epi cells.

Based on NMF clustering, we defined new glycolysis-related smooth muscle cell (SMC) and tissue stem cell (MSC) subtypes, including IER3+SMC, Unclear-Gly-SMC, ME1+MSC, and DDIT4+MSC (Figure 4A). KEGG enrichment analysis revealed that these newly defined subpopulations were mainly related with the TNF signaling pathway and IL-17 signaling pathway (Figure 4B). Metabolic activity analysis showed that IER3+SMC and ME1+MSC cells exhibited higher metabolic activity compared to other cells (Figure 4C). SCENIC analysis revealed that the transcription factor activity of these cells varied, potentially participating in different biological functions (Figure 4D). To explore the impact of these subpopulations on malignant epithelial cells, this research conducted cell communication analysis. The findings showed that the glycolysis-high IGFBP3+Epi and LHX9+Epi subpopulations communicated with all newly defined T cell subpopulations, compared to other malignant subpopulations (Figure 4E). By analyzing the ligand-receptor pairs involved in intercellular signaling, we observed significant differences in the communication probabilities and significance between various cell types. In the same cell type, there were more ligand-receptor pairs with higher activity. We also identified specific ligand-receptor complexes that regulate various immune-modulatory pathways coordinated by different immune cell subtypes. PPIA-BSG was the most active ligand-receptor pair. The interaction between PPIA and BSG not only promotes tumor cell invasion as well as metastasis but may also optimize the TME by impacting extracellular matrix remodeling, thus creating conditions for immune escape (Figures 4F–J).

### 3.5 Construction of a prognostic model based on high glycolysis epithelial cell subpopulation characteristic genes

**FIGURE 5:**
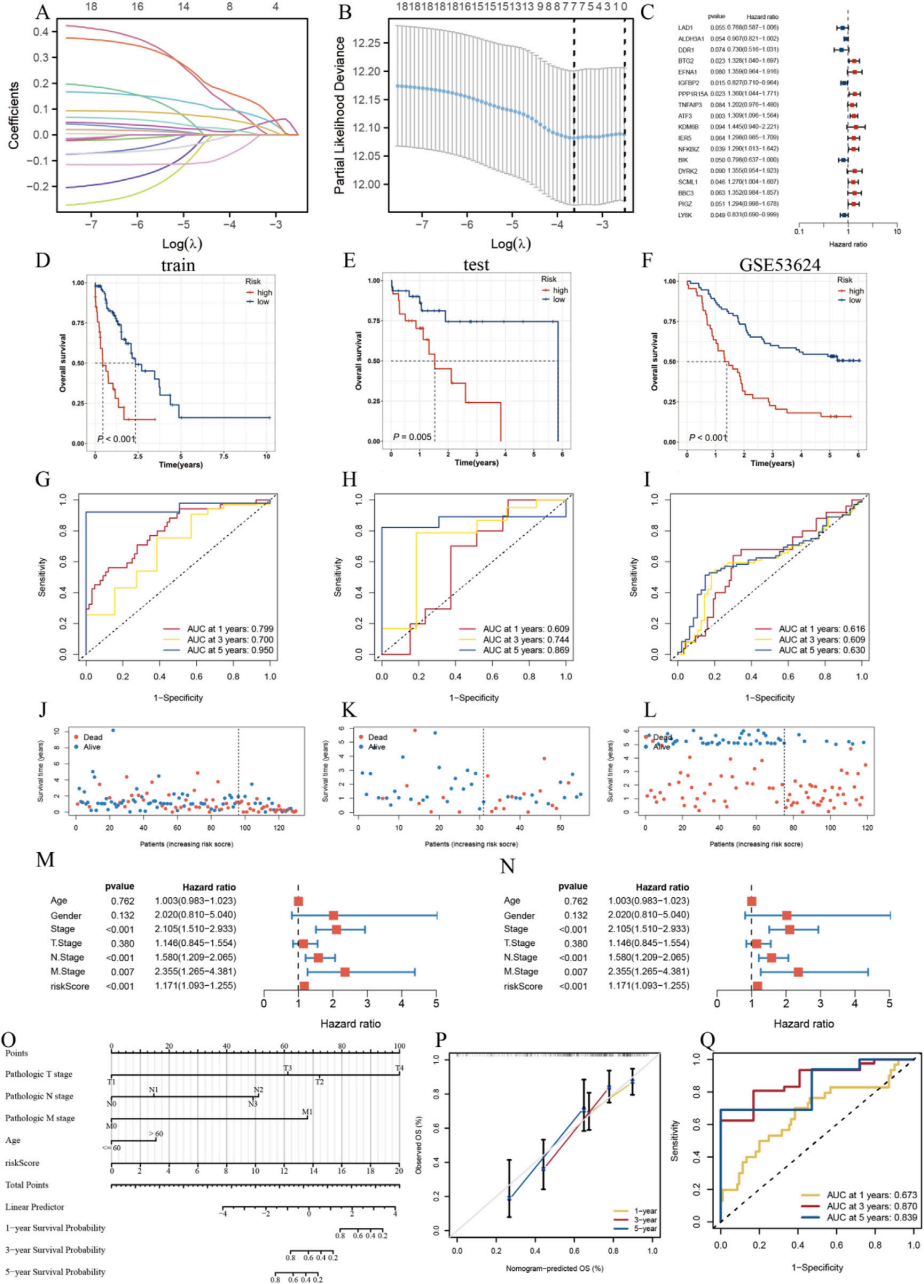
Construction of a Prognostic Model Based on Glycolysis-Related Feature Genes in the Epithelial Cell Subpopulation. **(A)**. Univariate Cox regression analysis of 18 genes to identify 7 hub genes with significant prognostic impact. **(B)**. Feature gene selection using LASSO regression model. **(C)**. Univariate Cox forest plot showing the hazard ratios of 7 hub genes. **(D–F)**. Kaplan-Meier survival curves for the training, test, as well as GSE53624 cohorts. **(G–I)**. ROC curves for the training, test, and GSE53624 cohorts. **(J–L)** Survival status of patients in the training, test, and GSE53624 cohorts. **(M)** Univariate Cox forest plot. **(N)** Multivariate Cox forest plot. **(O)** Nomograms based on patients’ clinical characteristics and risk scores. **(P)** Calibration curves provided validation of the model predictions for 1-, 3-, and 5-year survival predictions. **(Q)** Nomogram ROC Curve assesses the diagnostic performance of the nomogram.

To investigate the impact of glycolysis on patient prognosis, we calculated the glycolysis scores of the IGFBP3+Epi subpopulation with the highest glycolysis scores and used the LASSO regression model to select prognostic feature genes. First, the TCGA-ESCA cohort was split into training as well as testing cohorts in a 7:3 ratio. A univariate Cox regression model was employed to analyse the prognostic value of each IGFBP3+Epi subpopulation feature gene, resulting in 18 prognostic-related genes (P < 0.05) for further analysis: LAD1, ALDH3A1, DDR1, BTG2, EFNA1, IGFBP2, PPP1R15A, TNFAIP3, ATF3, KDM6B, IER5, NFKBIZ, BIK, DYRK2, SCML1, BBC3, PIGZ, and LY6K. Based on LASSO as well as multivariate Cox regression analysis, 7 risk genes were further chosen to build a risk model (Figures 5A, B). The risk score formula is: Risk score = (−0.5240 * IGFBP2(exp)) + 0.3094 * NFKBIZ(exp) + 0.2209 * ATF3(exp) + (−0.4890 * BIK(exp)) + 0.5311 * BTG2(exp) + 0.2400 * SCML1(exp) + (−0.1919 * LY6K(exp)). According to the optimal cutoff point from the survival curve, patients were split into high-risk as well a low-risk groups for survival analysis. This research carried out univariate Cox regression analysis on 18 genes and identified 7 hub genes with a remarkable impact on patient prognosis (Figure 5C).

The results showed that in the training (Figure 5D), testing (Figure 5E), and external validation GSE53624 (Figure 5F) cohorts, the OS of the low-risk group was remarkably better than that of the high-risk one. ROC curves indicated that the model’s risk score had good predictive ability for 1, 3, and 5-year OS in ESCC patients (Figures 5G–I). With the rise of risk score, the mortality rate of ESCC patients also elevated (Figures 5J–L). To further assess the predictive capacity of the model for ESCC patient prognosis, this research carried out univariate and multivariate Cox regression analyses combined with clinical data, finding that the risk score was an independent risk factor influencing patient prognosis (Figures 5M, N).

Based on these prognostic factors, we developed a Nomogram-based prognostic risk model (Figure 5O). This model accurately predicts 1-year, 3-year, and 5-year OS (Figure 5P), with corresponding AUC values of 0.756, 0.753, and 0.705, respectively (Figure 5Q).

### 3.6 Detection of the expression of proteins encoded by the genes in ESCC

**FIGURE 6:**
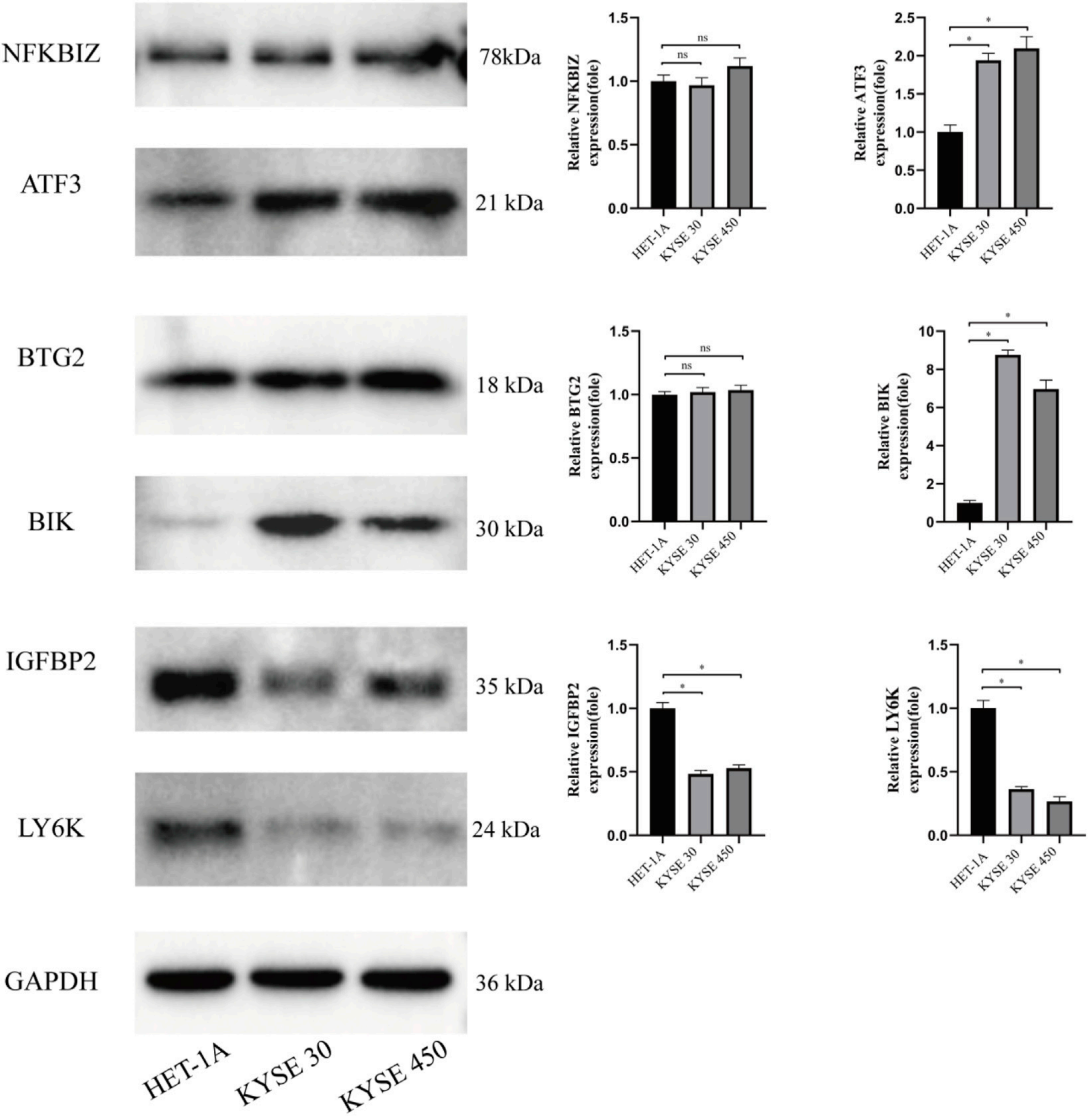
Expression of prognostic genes. The expression of NFKBIZ, ATF3, BTC2, BIK, CEBPB2, and LY6K proteins in normal esophageal epithelial cells (HET-1A) and esophageal cancer cells (KYSE30 and KYSE450). (*P < 0.05).

Western blot analysis revealed differential expression of target proteins in HET-1A, KYSE30, and KYSE450 cells: ATF3 protein was significantly upregulated in cancer cells; BIK expression was elevated in KYSE30 and KYSE450 cancer cells; IGFBP2 expression was lower in cancer cells compared to HET-1A cells, and LY6K expression was reduced in cancer cells; BTG2 protein did not show significant differences between normal and cancer cells (Figure 6).

### 3.7 Transcriptomic features of patients with different risk scores

**FIGURE 7:**
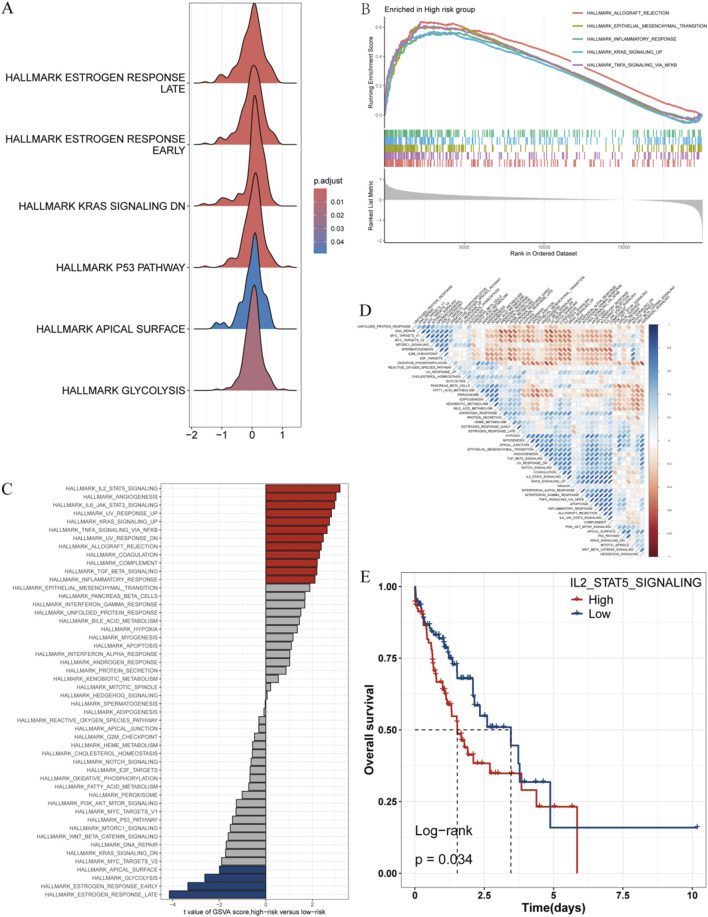
Transcriptomic Features of Patients with Various Risk Scores. **(A)** Ridge plot displaying HALL_MARKER enrichment in the low-risk group. **(B)** GSEA analysis displaying HALL_MARKER enrichment in the high-risk group. **(C)** Variations in hallmark pathway activity between the high-risk and low-risk groups as assessed via GSVA scores. **(D)** Correlation between risk scores and hallmark pathway activity evaluated via GSVA. **(E)** Kaplan-Meier survival curve displaying the remarkable correlation between OS and IL2_STAT5 GSVA scores.

To explore the molecular mechanisms underlying the correlation between risk scores and ESCC prognosis, this research carried out functional enrichment analysis. In the GSEA analysis based on HALL_MARKER, this research observed the low-risk group was enriched in early and late estrogen response, as well as p53 and KRAS signaling pathways (Figure 7A), while the high-risk one was enriched in epithelial-mesenchymal transition, inflammatory response, as well as TNF signaling pathways (Figure 7B). GSVA analysis showed that the high-risk group exhibited stronger activity in pathways related to TL2_STAT5_SIGNALING, angiogenesis, as well as IL6_JAK_STAT3_SIGNALING, whereas the low-risk group showed stronger activity in pathways related to ESTROGEN_RESPONSE_EARLY as well as ESTROGEN_RESPONSE_LATE (Figure 7C). Correlation analysis between risk scores and pathway scores further confirmed these findings (Figure 7D), indicating that risk scores have a close bearing on cancer-related biological processes and estrogen-related pathways.

We found pathways positively correlated with risk scores, like IL2_STAT5_SIGNALING, were associated with poor prognosis (Figure 7E). These findings imply the varying prognostic outcomes seen in the risk subgroups may be influenced by the activation or inhibition of these pathways.

### 3.8 Immune landscape between high-risk and low-risk groups

**FIGURE 8:**
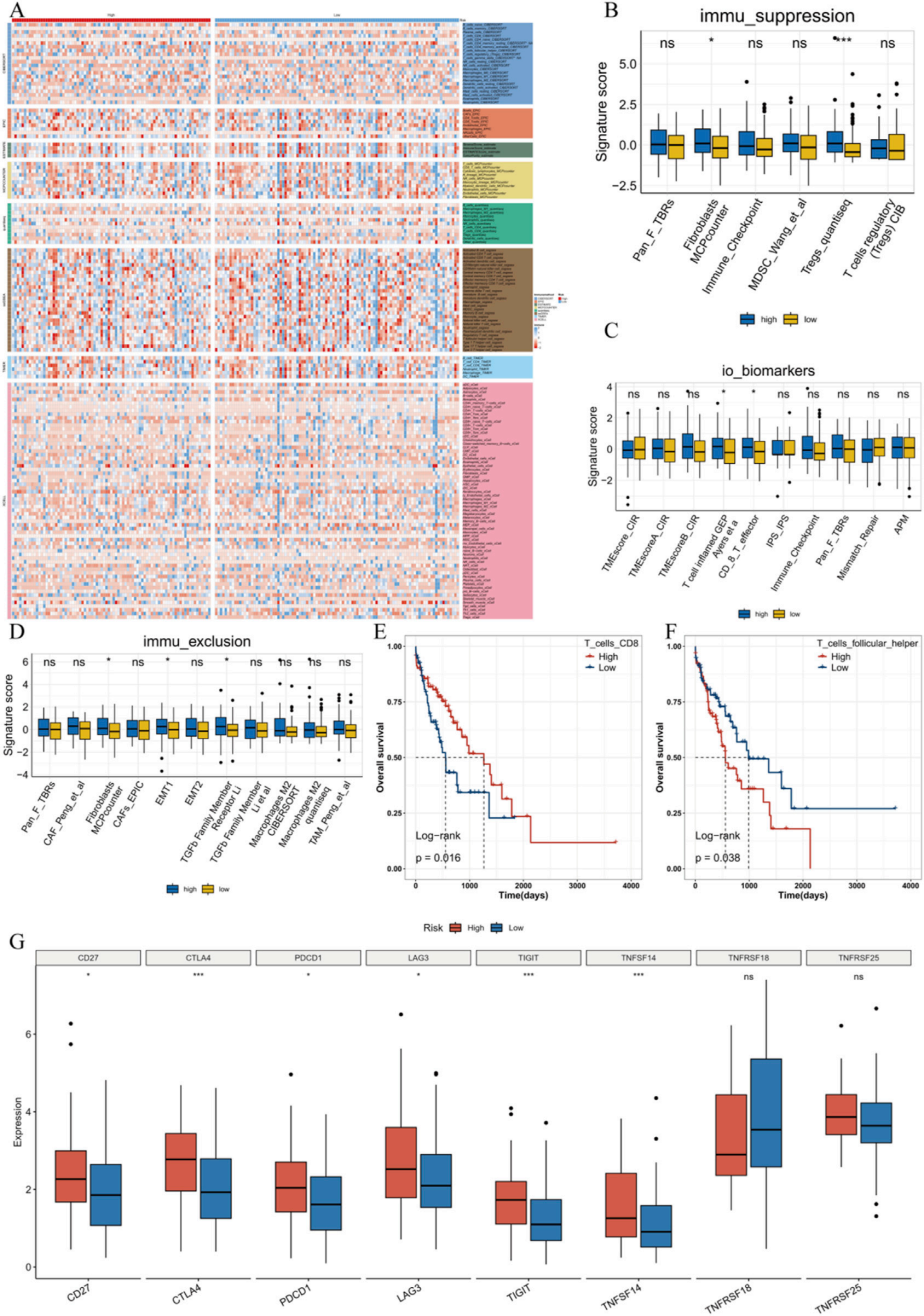
Immune landscape between high-risk and low-risk groups. **(A)** Immune infiltration abundance between high-risk and low-risk groups evaluated using eight immune infiltration assessment methods. **(B)** Distribution of immune suppression features between high-risk and low-risk patients. **(C)** Distribution of immunotherapy biomarkers between high-risk and low-risk patients. **(D)** Distribution of immune suppression features between high-risk and low-risk patients. **(E)** Survival curve based on CD8 T cell infiltration scores. **(F)** Survival curve based on helper T cell infiltration scores. **(G)** Boxplot of immune checkpoint expression.

Based on eight immune infiltration evaluation methods (CIBERSORT, ESTIMATE, quanTIseq, TIMER, IPS, MCPCounter, xCell, EPIC), this research analysed the variations in immune cell infiltration between high-risk and low-risk groups, and showed the findings in a heatmap (Figure 8A). Subsequently, through the IOBR package, we also presented the variations between high-risk and low-risk groups in immune suppression, immunotherapy, and immunotherapy biomarkers. The results suggested many immune-related feature scores were higher in the high-risk group (Figures 8B–D). For example, in the Fibroblasts MCPcounter and Tregs_quantised groups, there was a statistically significant difference between the high-risk and low-risk groups (P < 0.05), indicating that the high-risk group might lead to stronger immune suppression. In the immunotherapy biomarkers feature scores, significant differences were observed for certain markers (e.g., T cell inflamed GEP, CD8_T_effector) between the high-risk and low-risk groups, suggesting these markers may be associated with group differences or immune status. Regarding immune exclusion feature scores, most feature modules showed no remarkable variations between the two groups. However, stronger immune exclusion was observed in the high-risk group in the Fibroblasts MCPcounter, EMT1, and TGFb Family Member Receptor Li groups. To study the effect of immune cell infiltration on OS in ESCC patients, this research spilt patients into high and low immune infiltration groups built upon the median immune cell infiltration score derived from CIBERSORT. We found that low infiltration of CD8^+^ T cells significantly affected OS in ESCC patients, while high infiltration of T helper cell subsets involved in humoral immunity significantly influenced OS (Figures 8E, F). Furthermore, immune checkpoint analysis revealed that most immune checkpoints were expressed at higher levels in the high-risk group (Figure 8G).

### 3.9 Drug sensitivity analysis of high-risk and low-risk patients

**FIGURE 9:**
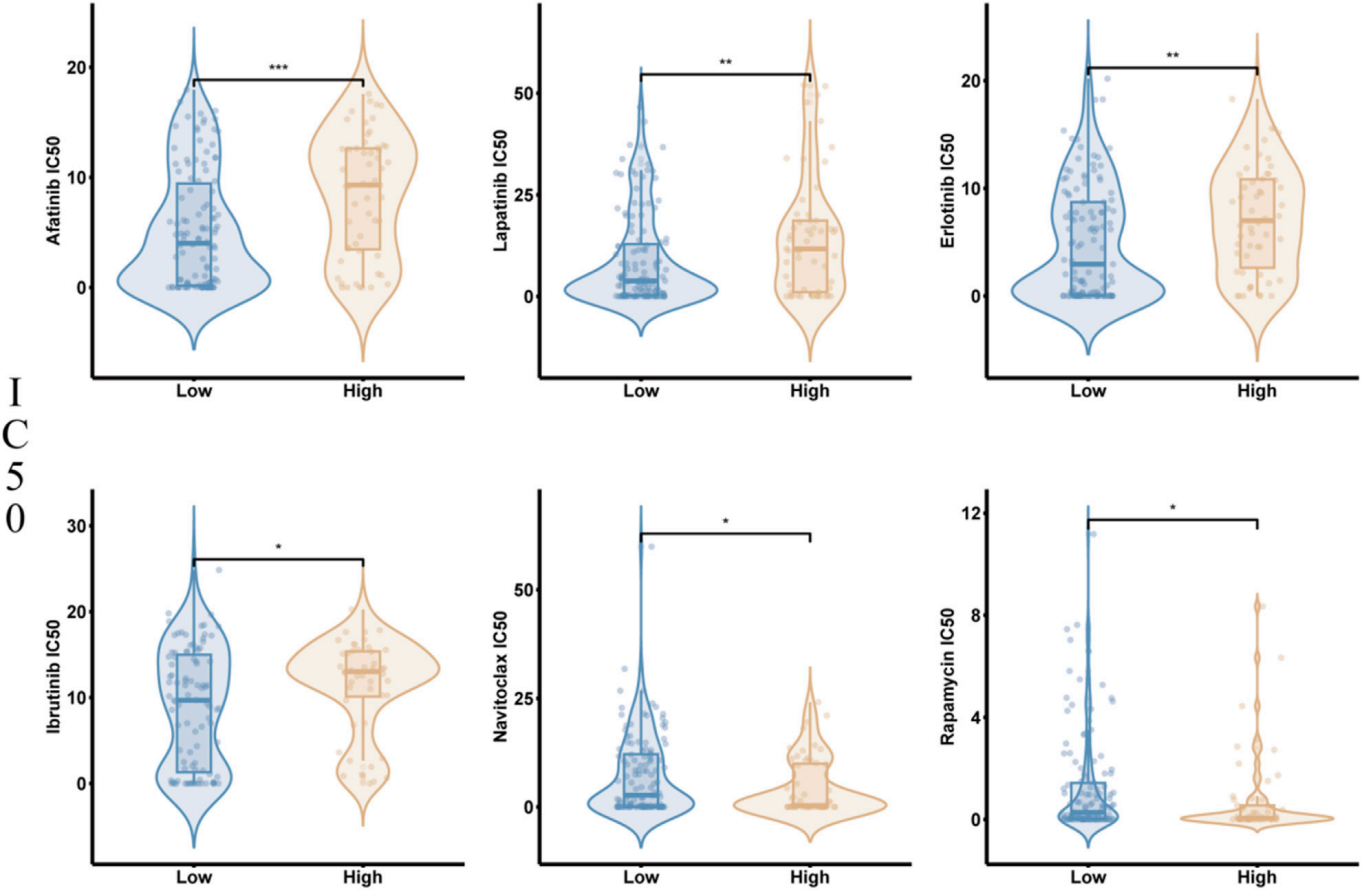
Drug Sensitivity Analysis Between High- and Low-Risk Patient Groups. The IC50 values of Afatinib, Lapatinib, Erlotinib, Ibrutinib, Navitoclax, and Rapamycin in high-risk and low-risk patient groups.

We investigated the sensitivity of ESCC patients in different risk groups to drugs such as Afatinib, Lapatinib, Erlotinib, Ibrutinib, Navitoclax, and Rapamycin. The findings suggested the high-risk group had remarkably higher IC50 values for Afatinib, Lapatinib, Erlotinib, as well as Ibrutinib compared to the low-risk one (P < 0.05). In contrast, the IC50 values for Navitoclax and Rapamycin were remarkably lower in the high-risk one compared to the low-risk one (P < 0.05). This indicates high-risk patients are more sensitive to Navitoclax as well as Rapamycin, while low-risk ones are more sensitive to Afatinib, Lapatinib, Erlotinib, and Ibrutinib (Figure 9).

## 4 Discussion

ESCC remains a remarkable global health challenge, especially in advanced stages, where treatment options are limited and prognosis is poor. Current therapeutic strategies often fail to fully consider the metabolic heterogeneity of tumors, especially the crucial role of glycolysis in tumor progression. This study highlights the importance of integrating metabolic profiling, particularly glycolytic activity, into clinical decision-making for ESCC, as it may help improve patient outcomes.

This study is based on single-cell transcriptomics from samples of 8 ESCC and 1 adjacent normal tissue, utilizing single-cell sequencing to analyze the glycolysis-related cell populations in ESCC. The results show that the glycolysis score in tumor tissues is remarkably higher than that in adjacent normal tissues. Within the tumor tissues, epithelial cells, monocytes, smooth muscle cells, and tissue stem cells exhibit higher glycolysis scores, with epithelial cells showing the most prominent increase, significantly higher than other cell types. The pathogenesis of ESCC typically involves a progression from normal epithelium to epithelial dysplasia, ultimately developing into invasive cancer. Based on the thickness of the atypical squamous cell replacement in the epithelium, epithelial dysplasia can be categorized into low-grade intraepithelial neoplasia as well as high-grade intraepithelial neoplasia (Nagtegaal et al., 2020). When the lesion further invades the lamina propria, it progresses into invasive cancer (Shimizu et al., 2009). Furthermore, the development of ESCC is associated with various risk factors. Garavello’s study found that in Italy and Switzerland, after adjusting for smoking and alcohol consumption, individuals with a family history of esophageal cancer among first-degree relatives had a 3.2 times higher likelihood of developing the disease compared to the general population (Garavello et al., 2005). Research has shown that long-term exposure of non-malignant esophageal epithelial cells to mainstream smoke extract or side-stream smoke extract induces phenotypic changes, gradually acquiring tumorigenic characteristics (Kim et al., 2010). Stromal fibroblasts is also crucial in the occurrence as well as progression of ESCC. Fibroblasts are the main components of the tissue stroma and have a key part in maintaining the balance of normal epithelial cells (Kalluri, 2016). Yamei Chen et al. carried out scRNA-seq and spatial transcriptomics on 79 samples from 29 ESCC patients, including tumor and adjacent normal (NOR), LGIN, and HGIN samples. They found ANXA1 is the ligand for fibroblast formyl peptide receptor 2 (FPR2), which helps maintain the balance of fibroblasts (Chen et al., 2023). Loss of ANXA1 causes uncontrolled transformation of normal fibroblasts into cancer-associated fibroblasts, with TGF-β secreted by malignant epithelial cells accelerating this transformation process. This mechanism suggests that esophageal malignant epithelial cells not only enhance their proliferative capacity through their own glycolytic activity but also promote cancer progression through interactions with fibroblasts. Therefore, the glycolytic activity of epithelial cells may be one of the key driving forces in the development of ESCC, providing new insights for further research and treatment.

To further investigate the prognostic role of glycolysis in ESCC, this study identified seven risk genes based on the characteristic genes of the high-glycolysis epithelial cell subpopulation and constructed a risk score model. The formula for the model is: Risk score = (−0.5240 * IGFBP2(exp))+ 0.3094 * NFKBIZ(exp) + 0.2209 * ATF3(exp) + (−0.4890 * BIK(exp)) + 0.5311 * BTG2(exp) + 0.2400 * SCML1(exp) + (−0.1919 * LY6K(exp)). The predictive results of this model showed patients in the high-risk group had remarkably poorer OS compared to those in the low-risk one, and the model showed strong predictive performance across multiple cohorts. With the rise in the risk score, the mortality rate of ESCC patients also elevated.

In current clinical practice, prognosis evaluation of ESCC primarily depends on TNM staging, which has limitations in accurately reflecting tumor metabolic heterogeneity and individual patient outcomes. Our study constructed a prognostic Nomogram integrating TNM staging, age, and glycolysis-related molecular features, significantly improving the precision of individualized survival prediction. Time-dependent ROC curves demonstrated that the Nomogram model exhibited robust predictive performance for 1-year, 3-year, and 5-year survival outcomes. Calibration curves further indicated strong concordance between predicted and observed survival rates, validating the model’s reliability. This comprehensive model enables clinicians to more accurately assess patient-specific survival risk, facilitating tailored therapeutic strategies and optimized clinical decision-making in ESCC management.

Additionally, Gene Set Variation Analysis (GSVA) revealed the high-risk group displayed stronger activation of pathways such as IL2_STAT5 signaling, angiogenesis, and IL6_JAK_STAT3 signaling, while the low-risk one suggested increased activation of pathways like estrogen response early and late. These findings suggest a close connection between the glycolysis risk score and TME. Further analysis in this study revealed differential glycolytic characteristics across various cell subpopulations. Glycolysis provides critical energy for the rapid proliferation of tumor cells and generates key metabolic intermediates, which are essential for the synthesis of nucleotides, lipids, as well as amino acids. These metabolic products, such as lactate, can accumulate and alter the tumor microenvironment, contributing to immune evasion and invasive behavior of the tumor. Furthermore, the accumulation of glycolytic products, such as lactate, can alter the tumor microenvironment, thereby promoting immune evasion and invasive behaviors of the tumor. Through the application of Non-negative Matrix Factorization (NMF) dimensionality reduction and KEGG pathway enrichment analysis, we identified five malignant epithelial cell subpopulations associated with glycolysis. Among these subpopulations, IGFBP3+Epi and LHX9+Epi cells exhibited higher glycolytic and metabolic activities. KEGG enrichment analysis further revealed the primary pathways in these subpopulations are closely associated with the ribosome, lysosome, and p53 signaling pathways. In the T-cell subpopulations, the transcription factor activity in CXCR4+CD8, DDIT4+Naïve, and CXCR4+Th17 subgroups was remarkably higher than in other cell groups. Additionally, the metabolic activity in smooth muscle cells and tissue stem cells, specifically in the IER3+SMC and ME1+MSC subpopulations, was also notably elevated. These findings deliver new insights into the part glycolysis plays in different cell subpopulations within esophageal squamous cell carcinoma and offer a strong theoretical foundation for future targeted therapeutic strategies. Furthermore, the accumulation of glycolytic products, such as lactate, can alter the tumor microenvironment (Chen et al., 2022; Lin et al., 2024), thereby promoting immune evasion and invasive behaviors of the tumor (Reuss et al., 2021; Lin et al., 2023; Qian et al., 2021). Through the application of Non-negative Matrix Factorization (NMF) dimensionality reduction and KEGG pathway enrichment analysis, we identified five malignant epithelial cell subpopulations associated with glycolysis. Among these subpopulations, IGFBP3+Epi and LHX9+Epi cells exhibited higher glycolytic and metabolic activities. KEGG enrichment analysis further showed the primary pathways in these subpopulations are closely associated with the ribosome, lysosome, and p53 signaling pathways. In the T-cell subpopulations, the transcription factor activity in CXCR4+CD8, DDIT4+Naïve, and CXCR4+Th17 subgroups was remarkably higher than in other cell groups. Additionally, the metabolic activity in smooth muscle cells and tissue stem cells, specifically in the IER3+SMC and ME1+MSC subpopulations, was also notably elevated. These findings provide new insights into the role of glycolysis in different cell subpopulations within esophageal squamous cell carcinoma and offer a strong theoretical foundation for future targeted therapeutic strategies.

In recent years, immune cells within the TME have been recognized as playing a crucial part in tumor biology and the sensitivity to anti-cancer therapies. Immune checkpoint blockade-based immunotherapy has emerged as a promising new adjuvant strategy for the treatment of esophageal squamous cell carcinoma (ESCC) (Liu et al., 2023). In certain cancer types, tumor cells evade immune detection by binding to the PD-L1 ligand, thereby inhibiting the activation of CD8^+^ T cells, which is a typical immune evasion mechanism. Furthermore, when CD8^+^ T cells enter a state of exhaustion, tumor malignancy progression is also aggravated (Iwai et al., 2002). This study further reveals that low infiltration of CD8^+^ T cells significantly impacts the OS of ESCC patients, while high infiltration of helper T cell subpopulations involved in humoral immune responses (such as Th1 and Th17 cells) also significantly affects patient prognosis. CD8+ T cells, as the main effector T cells, have a key part in anti-tumor immunity. However, the tumor microenvironment (TME) of ESCC often exhibits an immunosuppressive state, primarily due to the extensive infiltration of exhausted CD8+ T cells (Dinh et al., 2021; Chen et al., 2021). These exhausted CD8+ T cells are critical in tumor immune evasion and progression, leading to the failure of immune surveillance and allowing the tumor to escape immune system clearance. Therefore, a glycolysis-related immune risk score not only reflects the metabolic status of ESCC patients but also reveals the state of immune cells in their TME, further guiding the selection of clinical immunotherapies. By optimizing immunotherapy strategies, particularly through the combination of immune checkpoint inhibitors and glycolysis modulation therapies, it is hoped that the prognosis and survival rates of ESCC patients can be improved.

Using Western blot (WB) technology, this study validated the expression of the 7 key genes in the risk model in ESCC tissues. It was found that ATF3, and BIK were significantly overexpressed in tumor tissues, suggesting that these proteins may be involved in tumorigenesis and progression, particularly related to tumor cell proliferation, apoptosis inhibition, or microenvironment regulation. ATF3, a member of the ATF/CREB transcription factor family, is an important transcription factor induced by endoplasmic reticulum stress (ERS) responses. It is also involved in glucose and lipid metabolism and have a key part in tumor initiation and progression. Recent studies show that overexpression of ATF3 can counteract the effects of bortezomib on ESCC cell proliferation, apoptosis, as well as metabolic reprogramming. And ATF3 specifically binds to lactate dehydrogenase A (LDHA), inhibiting LDHA-mediated metabolic reprogramming and enhancing cellular response to bortezomib treatment (Chen et al., 2024). RT-qPCR analysis further demonstrated that ATF3 is highly expressed in ESCC cell lines, and knockdown of ATF3 significantly inhibits the migration capacity of TE-1 cells (Yang J. et al., 2024). Furthermore, ATF3, as a tumor suppressor gene, is underexpressed in ESCC tissues and negatively correlates with tumor cell proliferation, migration, and invasion (Xie et al., 2014). Its underlying mechanisms involve suppressing MMP-2 expression via the MDM2-mediated degradation pathway, reducing tumor vascular invasion, and enhancing cisplatin chemotherapy sensitivity. BIK is a pro-apoptotic protein belonging to the Bcl-2 family and plays an important role in regulating the mitochondrial-mediated intrinsic apoptosis pathway (Mebratu et al., 2023). Although studies on BIK in ESCC are limited, one study reported that a 16-gene signature, including BIK, generated using the Lasso model, could accurately predict the prognosis of ESCC (Ma and Luo, 2022). Additionally, LY6K and IGFBP2 have been implicated in ESCC progression. LY6K, a cancer-testis antigen, undergoes epigenetic activation in ESCC tissues and is proven to facilitate immune evasion (Guo et al., 2022). A peptide vaccine derived from LY6K (LY6K-177) exhibited favorable immunogenicity in a Phase I clinical trial, inducing CD8+ T cell-specific cytotoxicity against LY6K-expressing ESCC cells (Ishikawa et al., 2014). LY6K may represent a novel target for ESCC immunotherapy, particularly in combination with immune checkpoint inhibitors (e.g., PD-1/PD-L1 inhibitors). IGFBP2 has also attracted attention as a prognostic marker in ESCC. Elevated serum IGFBP2 levels correlate with poor prognosis in ESCC patients (Chengyun et al., 2022). IGFBP2 may promote tumor progression via the insulin-like growth factor (IGF) signaling pathway, although the precise molecular mechanisms warrant further exploration. Collectively, ATF3, BIK, LY6K, and IGFBP2 not only serve as key drivers in ESCC initiation and progression but also represent promising biomarkers and therapeutic targets. Their roles in tumor metabolism, apoptosis regulation, immune evasion, and treatment response merit further investigation. Future personalized therapeutic strategies targeting these molecules could potentially improve the prognosis of ESCC patients.

Finally, through drug sensitivity analysis, the research found high-risk group patients were more sensitive to chemotherapy drugs like Navitoclax as well as Rapamycin, while low-risk one patients displayed higher sensitivity to Afatinib, Lapatinib, Erlotinib, and Ibrutinib. This finding suggests that the glycolysis-related risk score not only predicts patient prognosis but may also deliver guidance for developing personalized treatment plans. Furthermore, this result indicates remarkable variations in chemotherapy drug sensitivity between different risk groups, which may be closely related to their molecular characteristics and metabolic states. In the high-risk group, glycolysis levels are typically elevated, a metabolic state that may promote the expression of anti-apoptotic proteins like Bcl-2. Navitoclax, by inhibiting these anti-apoptotic proteins (Harrison et al., 2022), can disrupt the metabolic advantage and survival dependency of tumor cells, significantly enhancing its cytotoxic effect on cancer cells. Rapamycin, an mTOR signaling pathway inhibitor, and other rapalogs can directly target precancerous cells and delay organismal aging, thus slowing cancer development (Blagosklonny, 2023). In the high-risk group, glycolysis levels are abnormally elevated, and mTOR signaling is a key regulatory factor in glycolytic metabolism. By inhibiting mTOR, Rapamycin can effectively block tumor cell dependence on glycolysis in the high-risk group, inducing metabolic inhibition as well as cell death. This research hypothesize, when used in combination, these two drugs may have a synergistic effect, especially in high-risk patients with aberrantly active glycolysis and enhanced anti-apoptotic signaling, thereby significantly improving treatment outcomes. On the other hand, the increased sensitivity of low-risk group patients to Afatinib, Lapatinib, Erlotinib, and Ibrutinib may be associated with the inhibition of the EGFR and BTK signaling pathways by these drugs. These results not only confirm the important value of glycolysis-related risk scores in predicting patient prognosis but also provide potential reference points for personalized treatment of patients in various risk groups. In the future, further clinical studies can verify the efficacy of these drugs in patients of different risk groups and optimize treatment strategies in combination with risk scores, thereby guiding more precise stratified treatment for ESCC patients and improving therapeutic outcomes.

Glycolysis inhibition has emerged as a promising therapeutic approach in cancer treatment. Studies have demonstrated that the combined inhibition of glycolysis and mitochondrial oxidative phosphorylation can trigger catastrophic energy depletion, thereby significantly suppressing tumor progression (Dong et al., 2022). As a pivotal pathway in cellular energy metabolism, glycolysis is closely associated with cancer cell drug sensitivity. Beyond serving as an energy source, glycolysis generates crucial metabolic intermediates, including lactate, ATP, NADH, and 2-phosphoglycerate. These intermediates modulate several intracellular signaling pathways, such as HIF-1α, mTOR, and PI3K/Akt, ultimately influencing tumor cell responses to chemotherapy and targeted therapies. Consequently, glycolysis plays a crucial role in regulating drug resistance mechanisms (Hayashi et al., 2019; Marcucci and Rumio, 2021; Zhou et al., 2012). In our glycolysis-associated risk model, the seven signature genes influence drug sensitivity through distinct signaling pathways. For instance, DDR1 is involved in the regulation of the Raf/MEK/ERK and PI3K/Akt pathways, thereby enhancing cellular sensitivity to chemotherapeutic agents (Chappell et al., 2020). Similarly, ATF3 is implicated in ferroptosis induction via inhibition of the Nrf2/Keap1/xCT pathway, which enhances cisplatin sensitivity in gastric cancer cells (Fu et al., 2021). Moreover, the metabolic state of the tumor microenvironment (TME), encompassing immune cells, stromal components, and vasculature, can affect therapeutic efficacy through glycolytic reprogramming (Liu et al., 2024). Thus, glycolysis not only provides energy and biosynthetic intermediates to support tumor cell proliferation but also exerts multifaceted effects on drug response by modulating signaling pathways, altering drug target expression, and reshaping the TME. Targeting glycolytic pathways holds significant potential as a strategy to enhance the efficacy of anticancer therapies. Moving forward, we aim to further investigate the mechanistic differences in chemotherapy sensitivity between different risk groups stratified by our model, ultimately facilitating the development of more precise, individualized treatment regimens for clinical application.

## 5 Conclusion

In conclusion, this research reveals the remarkable role of glycolysis-related cell subtypes in ESCC and establishes a glycolysis-related risk score model, providing a deep analysis of its substantial impact on patient prognosis. These findings not only offer new directions for future therapeutic interventions but also deepen our understanding of tumor metabolism and its microenvironment. However, there are certain limitations to this research, such as the need for further validation of these results in larger patient cohorts and a more detailed exploration of the specific functional mechanisms underlying these metabolic alterations. Further mechanistic studies focusing on the specific molecules incorporated in the model are needed to clarify their causal relationships with ESCC. Additionally, *in vitro* drug response experiments are warranted to elucidate the molecular basis underlying drug sensitivity differences between risk groups. The results of this research deliver a novel supplement to the existing ESCC research framework and lay the foundation for developing innovative therapeutic strategies targeting the metabolic vulnerabilities of the tumor microenvironment. Looking ahead, integrating single-cell transcriptomic data with clinical outcomes will not only optimize the selection of therapeutic targets but also open new pathways for improving the treatment and management of ESCC patients, while further advancing the development of precision medicine.

## Data Availability

The datasets presented in this study can be found in online repositories. The names of the repository/repositories and accession number(s) can be found in the article/supplementary material.
